# Development of a specific fluorescent phage endolysin for *in situ* detection of *Clostridium* species associated with cheese spoilage

**DOI:** 10.1111/1751-7915.12883

**Published:** 2017-11-21

**Authors:** Natalia Gómez‐Torres, Matthew Dunne, Sonia Garde, Rob Meijers, Arjan Narbad, Marta Ávila, Melinda J. Mayer

**Affiliations:** ^1^ Departamento de Tecnología de Alimentos Instituto Nacional de Investigación y Tecnología Agraria y Alimentaria (INIA) Carretera de La Coruña km 7 28040 Madrid Spain; ^2^ European Molecular Biology Laboratory (EMBL) Hamburg Outstation Notkestrasse 85 22607 Hamburg Germany; ^3^ Gut Health and Food Safety Institute Strategic Programme Quadram Institute Bioscience Colney Norwich NR4 7UA UK; ^4^ Present address: Institute of Food, Nutrition and Health ETH Zurich LFV B36, Schmelzbergstr. 7 8092 Zurich Switzerland

## Abstract

Late blowing defect (LBD) is a major cause of spoilage in cheeses, caused by the growth of *Clostridium* spp. in the cheese matrix. We investigated the application of CTP1L, a bacteriophage endolysin active against *Clostridium tyrobutyricum*, and its enzymatically active and cell wall‐binding domains (EAD and CBD) attached to green fluorescent protein (GFP) to detect dairy‐related *Clostridium* species by fluorescence microscopy. GFP‐CTP1L and GFP‐CBD demonstrated specificity for *Clostridium* spp. by labelling 15 and 17 of 20 *Clostridium* strains, respectively, but neither bound to other members of the cheese microbiota. However, GFP‐EAD did not label any *Clostridium* strain tested. Unexpectedly, GFP‐CTP1L and GFP‐CBD were also able to bind to clostridial spores. In addition, GFP‐CBD allowed us to visualize the vegetative cells of *C. tyrobutyricum* directly in the matrix of a LBD cheese. Site‐directed mutants of GFP‐CTP1L and GFP‐CBD were made to examine the amino acids involved in binding and oligomer formation. Oligomerization was not essential for binding, but specific mutations in the CBD which affected oligomer formation also affected binding and lytic activity. We conclude that GFP‐CTP1L and GFP‐CBD could be good biomarkers for rapid detection of *Clostridium* spores in milk, so measures can be taken for the prevention of LBD in cheese, and also provide effective tools to study the development of *Clostridium* populations during cheese ripening.

## Introduction

Butyric acid fermentation, also known as late blowing defect (LBD), is an important problem to the cheese industry and without appropriate controls can cause severe economic losses (Garde *et al*., 2013). It is a major cause of spoilage in semi‐hard and hard ripened cheeses, resulting in texture and flavour defects including the formation of irregular cracks in the cheese paste and a rancid taste. LBD is caused by the outgrowth of anaerobic, gram‐positive spore‐forming species of the genus *Clostridium*, capable of fermenting lactic acid with the production of butyric acid, acetic acid, carbon dioxide and hydrogen. *Clostridium tyrobutyricum* is considered the primary cause, but other species such as *Clostridium sporogenes*,* Clostridium beijerinckii* and *Clostridium butyricum* have also been shown to contribute (Klijn *et al*., 1995; Cocolin *et al*., 2004; Le Bourhis *et al*., 2005, 2007). *Clostridium* spores are ubiquitous, highly resistant to heat, chemicals, irradiation, pressure and desiccation and can persist in the environment for a long time. Traditional methods for detecting clostridia are based on spore germination and vegetative growth. Detecting *Clostridium* in dairy products is still a challenge: the commonly used most probable number techniques are time‐consuming, requiring long periods of incubations and fail to differentiate among spore formers at the species level. Molecular methods have overcome the limitations of cultivation‐based approaches to detect spoilage bacteria within 24 h in milk or dairy products using PCR (Herman *et al*., 1995, 1997; Klijn *et al*., 1995; Cocolin *et al*., 2004; Le Bourhis *et al*., 2007). More recently, real‐time PCR protocols have been developed for *C. tyrobutyricum* cell and spore enumeration in feed, faeces and dairy samples (Bassi *et al*., 2013) and for the simultaneous detection of *C. beijerinckii*,* C. sporogenes* and *C. tyrobutyricum* in milk samples (Morandi *et al*., 2015). PCR‐based detection has been studied because of its specificity, high sensitivity and avoidance of enrichment culturing. However, DNA extraction efficiency may be decreased by a wide range of substances coming from the cheese matrix itself (Bonaiti *et al*., 2006). Moreover, even if DNA yield is high, inhibitors may lower PCR sensitivity (Wilson, 1997). Few other non‐cultural methods, recently reviewed by Brändle *et al*. (2016), have been proposed to detect *C. tyrobutyricum*. Immunological approaches are considered very specific, but these methods have low sensitivity. As an alternative to antibody ligands, Lavilla *et al*. (2012) produced superparamagnetic beads coated with specific peptides to specifically bind *C. tyrobutyricum* spores for concentration of spores prior to detection.

The use of bacteriophage endolysins could be an alternative technique to detect spoilage *Clostridium* in milk and cheese. The applications of bacteriophages and their endolysins in food safety have been extensively reviewed (Gutiérrez *et al*., 2016; Schmelcher and Loessner, 2016). Bacteriophages are the most abundant self‐replicating entities on the planet and have been optimized by millions of years of evolution to specifically recognize and effectively kill their target cells (Hendrix, 2002). Endolysins are peptidoglycan hydrolases, encoded by the genomes of lytic phages and produced at the end of their multiplication cycle to lyse the host cell and release the phage progeny (Loessner, 2005; Fischetti, 2010). Endolysins from a gram‐positive phage‐host background are generally characterized by a modular architecture, featuring distinct enzymatically active domains (EAD) and cell wall‐binding domains (CBD). The EADs digest the cell wall peptidoglycan layer and can be classified based on their catalytic activity (muramidases, glucosaminidases, lytic transglycosylases, amidases or endopeptidases). The CBDs direct the endolysin to specific cell wall‐associated ligands with high specificity and strong binding affinity (Loessner, 2005; Schmelcher *et al*., 2010, 2011). The specificity of the CBD has been applied in bacterial detection systems, because it allows the direct targeting of bacteria while leaving the background flora unaffected (Kretzer *et al*., 2007; Schmelcher *et al*., 2010; Yu *et al*., 2016). Fusion of a bacteriophage endolysin CBD with green fluorescent protein (GFP) produces a fluorescent probe that is able to rapidly recognize and bind to the host cells of a given species or even to individual serovars (Loessner *et al*., 2002; Schmelcher *et al*., 2011). Endolysins are considered safe due to their highly conserved target sites and the limited number of possible resistance mechanisms (Schmelcher *et al*., 2012), leading to increasing interest in possible food safety applications. However, few studies have investigated the efficacy of phage endolysins in food products compared to reports on clinical applications (Schmelcher and Loessner, 2016). In addition, there is a shortage of studies on their use as a biocontrol strategy or as a detection system against spoilage bacteria like *C*. *tyrobutyricum*.

Mayer *et al*. (2010) isolated the endolysin of ΦCTP1, a bacteriophage infecting *C. tyrobutyricum*, and demonstrated *in vitro* its ability to lyse *C. tyrobutyricum* cells and control bacterial levels in buffer and in milk. In a recent study, a crystal structure of the activated endolysin CTP1L consisting of a complex between the full‐length protein and an N‐terminally truncated C‐terminal CBD was presented (Dunne *et al*., 2016). The main purpose of this study was to investigate the application of the CTP1L endolysin and its N‐ and C‐terminal domains, attached to GFP, to specifically detect *Clostridium* species responsible for LBD in cheese by fluorescence microscopy. Furthermore, site‐directed mutants of GFP‐CTP1L and GFP‐CBD were investigated to shed light on the interactions between endolysin structure and lytic and binding activities.

## Results

### CTP1L C‐terminal domain mediates endolysin binding to cells

**Figure 1:**
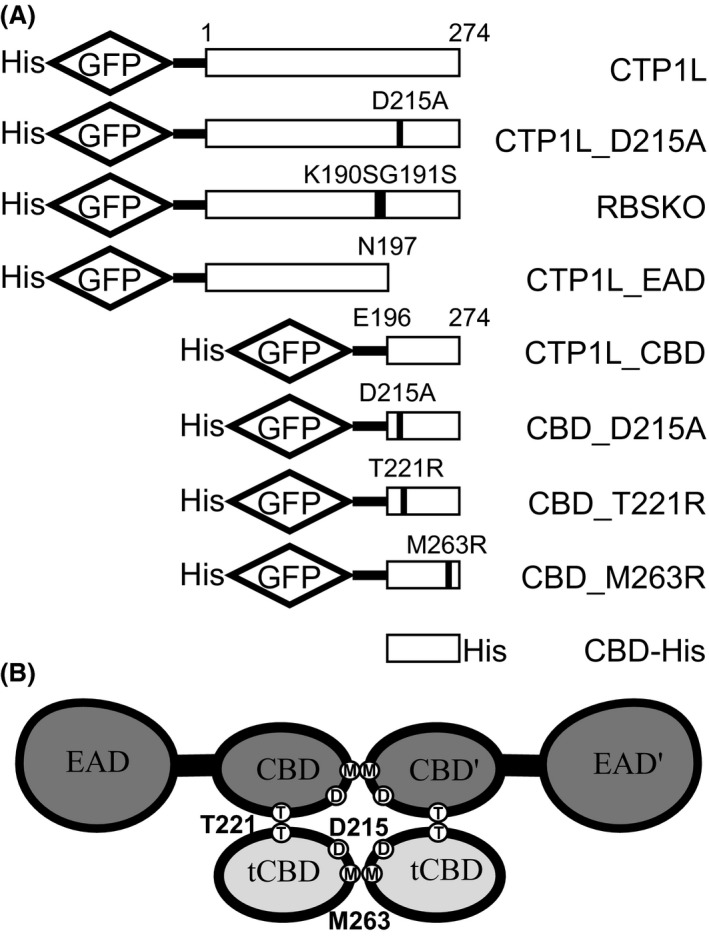
(A) Wild type and mutant constructs used in this study; (B) cartoon representation of the CTP1L heterotetrameric complex with the positions of key residues D215, M263 and T221 highlighted.

**Figure 2:**
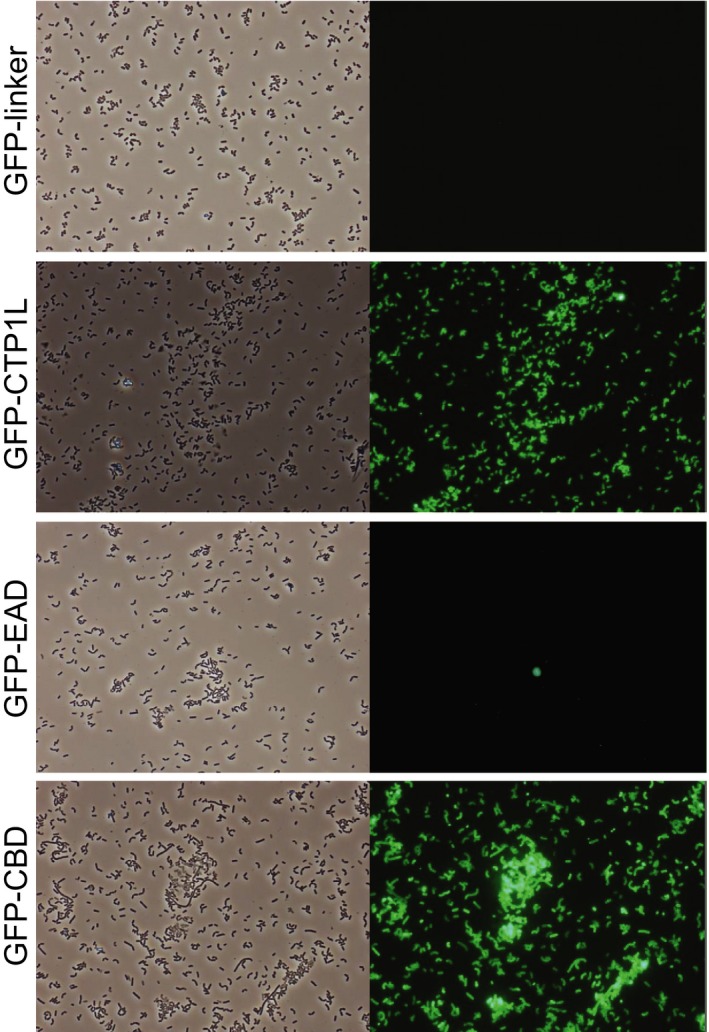
Cells of *C. tyrobutyricum* NCIMB 9582 after incubation with GFP‐labelled proteins at standard conditions of binding (37°C, 20 min in PBS‐T buffer). Pictures were taken with phase contrast (left) and green fluorescence (right) at a magnification of ×400.

The crystal structure of the full‐length CTP1L endolysin was recently solved by Dunne *et al*. (2016), showing an EAD (residues 1–190) connected by a linker of four residues to a CBD (residues 195–274), which was confirmed to bind to *C. tyrobutyricum*. To investigate the cell wall binding activity of the full‐length CTP1L and its CBD and EAD domains separately, constructs were N‐terminally tagged with GFP (Fig. 1) and tested with vegetative cells of *C. tyrobutyricum* NCIMB 9582. GFP‐CTP1L and GFP‐CBD both showed clear and strong binding to the entire surface of clostridial cells, while cells incubated with GFP‐EAD or the GFP‐linker control produced from pET15b were not labelled, although a faint outline of clostridial cells was visible due to native autofluorescence (Fig. 2).

When the binding ability of GFP‐labelled CTP1L and its CBD was assessed under different conditions, fluorescent labelling was found to be optimal with reaction conditions of 20 min at 37°C, although binding was also demonstrated using shorter incubation times (5, 10 min) and lower temperatures (30°C and room temperature, 20–25°C). Varying the NaCl concentration (2, 1, 0.5, 0.2 M) did not affect binding, but reducing the pH of the incubation buffer from 7.40 to 5.2 decreased fluorescence. GFP‐labelled proteins bound both exponential and stationary phase cells, but a higher fluorescence signal was observed for the latter. In addition, using boiled clostridial cells resulted in stronger binding of GFP‐endolysins than to fresh cells (Fig. S1).

### CTP1L binds specifically to *Clostridium* spp.

We investigated the binding of GFP‐CTP1L, GFP‐EAD and GFP‐CBD to vegetative cells of different clostridial species, most of them isolated from Manchego cheeses with LBD (Table 1). GFP‐CTP1L and GFP‐CBD bound to 15 and 17, respectively, of 20 *Clostridium* strains tested, including strains which had been demonstrated to cause LBD (Gomez‐Torres *et al*., 2015) and all the *C. tyrobutyricum* strains. However, GFP‐EAD and the GFP‐linker control did not bind to any *Clostridium* strain tested. Cell fluorescence was more intense for GFP‐CBD than for the full‐length GFP‐CTP1L binding in the case of *C. tyrobutyricum* CECT 4011 and INIA 69 strains. In addition, GFP‐CBD was able to bind to two *C. beijerinckii* strains (LMG 5716 and INIA 73) that did not show binding with GFP‐CTP1L.

**Table 1:** Binding of GFP endolysins to *Clostridium* strains.^a^

*Clostridium* strains	GFP‐CTP1L	GFP‐EAD	GFP‐CBD
*C. tyrobutyricum* NCIMB 9582	+++	−	+++
*C. tyrobutyricum* CECT 4011^b^	++	−	+++
*C. tyrobutyricum* INIA 68^b^	++	−	++
*C. tyrobutyricum* INIA 69	+	−	++
*C. tyrobutyricum* INIA 78	+	−	+
*C. tyrobutyricum* INIA 79	+	−	+
*C. sporogenes* ATCC 17886	+	−	+
*C. sporogenes* CECT 892	++	−	++
*C. sporogenes* INIA 71^b^	+	−	+
*C. beijerinckii* LMG 5716	−	−	++
*C. beijerinckii* INIA 73	−	−	+++
*C. beijerinckii* INIA 72	+++	−	++
*C. beijerinckii* INIA 63^b^	++	−	++
*C. beijerinckii* INIA 65	−	−	−
*C. beijerinckii* INIA 74	−	−	−
*C. beijerinckii* INIA 75	++	−	++
*C. beijerinckii* INIA 76	+++	−	+++
*C. beijerinckii* INIA 77	+++	−	+++
*C. butyricum* CECT 361	++	−	++
*C. butyricum* INIA 66	−	−	−

a +, low binding; ++, binding, +++, strong binding; −, no binding.

b Strains shown to cause LBD (Gomez‐Torres *et al*., 2015).

To study the level of specificity of GFP‐CTP1L and GFP‐CBD, binding assays were performed with lactic acid bacteria belonging to the cheese microbiota, including several commercial cheese cultures widely used in cheese manufacture. No fluorescence was observed by microscopy (Table 2), suggesting that these proteins have a genus level of specificity which makes them potentially useful for detecting *Clostridium* spp. in milk and cheese matrices.

**Table 2:** Binding of GFP endolysins to lactic acid bacteria and *Clostridium* spores.^a^

Binding target	GFP‐CTP1L	GFP‐CBD
*Lactococcus lactis* subsp. *lactis* INIA 415	−	−
*L. lactis* subsp. *cremoris* MG1363	−	−
*L. lactis* subsp*. lactis* NCIMB 700176	−	−
*Enterococcus faecalis* FI10734	−	−
*Enterococcus faecium* FI10735	−	−
*Lactobacillus reuteri* 1063N	−	−
Choozit™ MA 11	−	−
Choozit™ TA 54	−	−
FD‐DVS FLORA DANICA	−	−
RSF 636	−	−
*C. beijerinckii* INIA 77 spores	+	+
*C.tyrobutyricum* NCIMB 9582 spores	+	+
*C. tyrobutyricum* CECT 4011 spores	+	+
*C. tyrobutyricum* INIA 68 spores	+	+
*C. beijerinckii* INIA 75 spores	+	+
*C. butyricum* CECT 361 spores	+	+
*C. sporogenes* CECT 892 spores	+	+

a +, binding; −, no binding.

### CTP1L is able to bind to Clostridium spores

**Figure 3:**
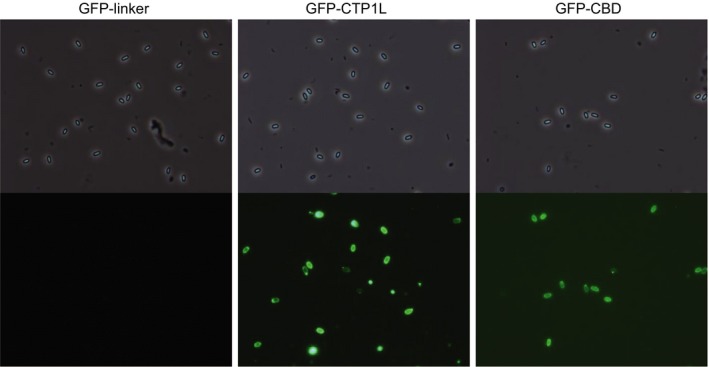
Fluorescent labelling of *C. beijerinckii* INIA 77 spores. Pictures were taken with phase contrast (top) and green fluorescence (bottom) at a magnification of ×1000.

We tested the ability of GFP‐CTP1L and GFP‐CBD to bind to spores of *Clostridium* spp. Although lysis and separation protocols were applied to get pure cultures of spores, we only obtained a culture without vegetative cells or their debris from *C. beijerinckii* INIA 77. Surprisingly, GFP‐CTP1L and GFP‐CBD were both able to label spores of *C. beijerinckii* INIA 77, while the GFP‐linker control did not bind (Fig. 3). Binding of GFP‐CTP1L and GFP‐CBD was also seen to spore preparations from several other clostridial species (Table 2).

### Effect of specific mutations on binding activity

**Figure 4:**
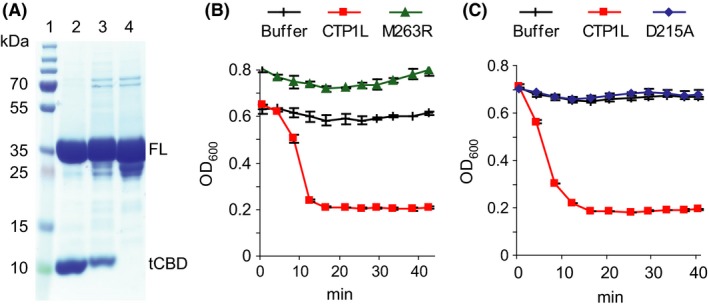
Effect of site directed mutations on oligomerization and lytic activity. A. SDS‐PAGE of Ni‐NTA‐purified endolysins showing copurification of tCBD, lane 1, Marker, lane 2, wild‐type CTP1L, lane 3, mutant CTP1L‐M263R, lane 4, mutant CTP1L‐D215A, FL, full‐length endolysin, tCBD, copurified free CBD domain; B. lytic activity of CTP1L‐M263R; C. lytic activity of CTP1L‐D215A. Results are the mean of duplicate assays ± SD.

In order to investigate the amino acids involved in endolysin binding, we made GFP‐labelled constructs of mutants of the wild‐type CTP1L and its CBD which had previously been shown to exhibit changes in CTP1L dimerization and lytic activity (Dunne *et al*., 2014; Dunne, 2014; Dunne *et al*., 2016; Fig. 1A). The wild‐type enzyme exists in solution as a heterotetrameric complex, consisting of two full‐length CTP1L proteins and two truncated CBDs (tCBD) expressed from an internal translational start site within the CTP1L gene (Fig. 1B; Dunne *et al*., 2016). Dunne *et al*. (2016) showed that two modes of dimerization, side‐by‐side (CBD to tCBD) and head‐on interaction (CBD to CBD and tCBD to tCBD), can exist between the four CBDs at the centre of the enzyme complex, with the activated endolysin consisting of a heterodimer with the side‐by‐side interaction. Mutation RBSKO (ribosomal binding site knock‐out) destroys the internal translation site that makes the tCBD, keeping the enzyme as a monomer and reducing lytic activity at low enzyme concentrations; this mutation can be partially rescued by the addition of free CBD (Dunne *et al*., 2016). Mutation T221R affects side‐by‐side binding of two CBD units; previous work showed that this mutation abolishes lytic activity and SDS‐PAGE analysis suggested that the mutant protein does not form a complex with tCBD (Dunne *et al*., 2014). Mutations M263R and D215A occur at the base of the CBD and were designed to prevent head‐on dimer formation. When His‐tagged wild‐type CTP1L is purified by Ni‐NTA chromatography, attached tCBDs are co‐purified then are separated from the complex by denaturing SDS‐PAGE (Dunne *et al*., 2016; Fig. 4A); gel analysis suggests that CTP1L‐M263R can still interact with its tCBD (Fig. 4A), but the mutant does not have lytic activity (Fig. 4B). D215A forms electrostatic interactions in the head‐on interface and also plays a role in interacting with the side‐by‐side dimer – its mutation was previously shown to convert CTP1L to a monomer but there is a minimal presence of oligomeric species (Dunne *et al*., 2014). We found that this mutation also renders the endolysin inactive (Fig. 4C).

**Figure 5:**
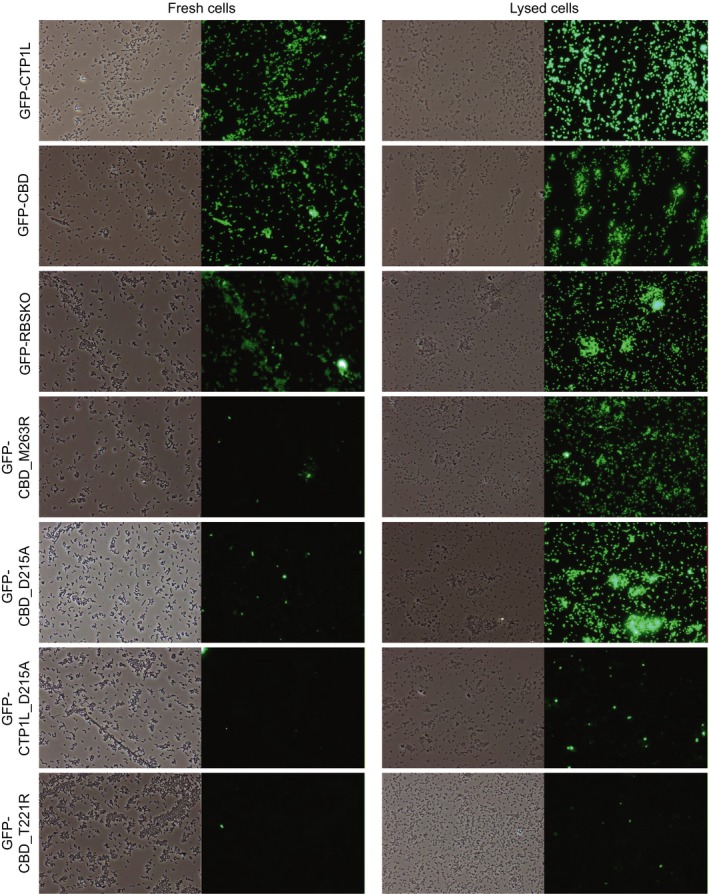
Impact of site directed mutations on binding to *C. tyrobutyricum* NCIMB 9582 fresh (left column) or lysozyme‐treated (right column) cells. Pictures were taken with phase contrast and green fluorescence at a magnification of ×400.

Mutant proteins fused to GFP were produced either as CBDs (GFP‐CBD_D215A, GFP‐CBD_M263R, GFP‐CBD_T221R) or as full‐length proteins (GFP‐CTP1L_D215A, GFP‐CTP1L_RBSKO). Each mutant construct was evaluated for binding to fresh vegetative cells of *C. tyrobutyricum* NCIMB 9582 in the stationary phase (Fig. 5). GFP‐RBSKO was the only mutant able to bind the *C. tyrobutyricum* cells to a similar extent as its counterpart GFP‐CTP1L. Proteins with M263R and D215A mutations displayed binding only to a few specific cells. Upon detailed observation under contrast phase microscopy, it appeared that only lysed cells were labelled by these mutants. To verify this observation, we treated *C. tyrobutyricum* NCIMB 9582 cells with lysozyme before the binding assay. In contrast to untreated cells, the vast majority of lysozyme‐treated cells were labelled with GFP‐CBD_D215A, GFP‐CBD_M263R mutants and, to a lesser extent, GFP‐CTP1L_D215A mutant (Fig. 5). We also observed more intensive binding of GFP‐CTP1L, GFP‐CBD and GFP‐RBSKO to lysozyme‐treated *C. tyrobutyricum* cells. In contrast, GFP‐CBD_T221R did not show binding to fresh or lysozyme‐treated cells.

**Figure 6:**
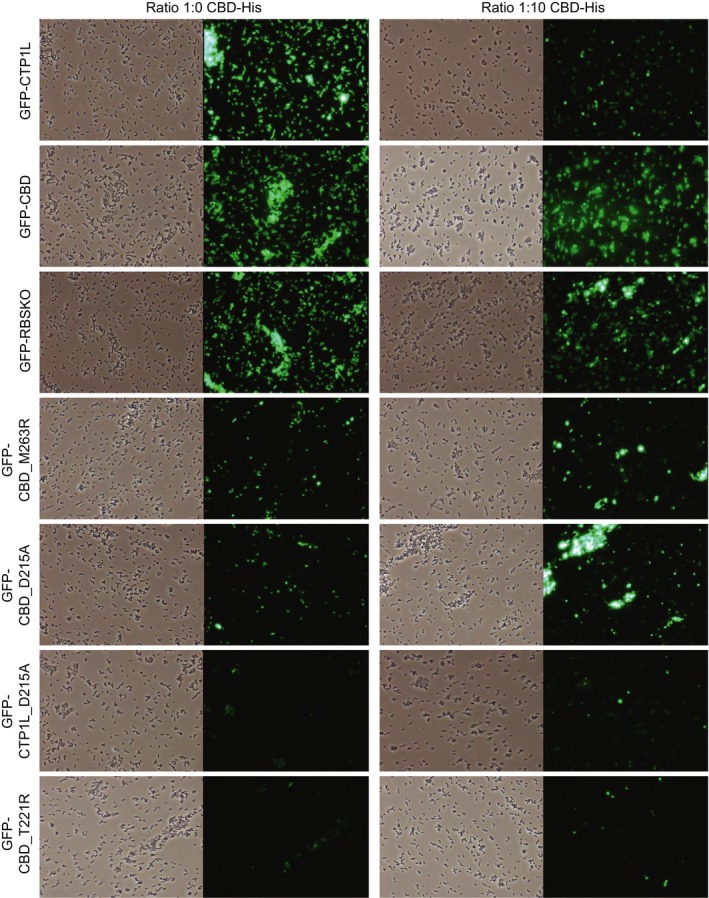
Fluorescence microscopy images of binding of wild type and mutant GFP‐endolysins to *C. tyrobutyricum* NCIMB 9582 with (right column) and without (left column) addition of free CBD‐His. Pictures were taken with phase contrast and green fluorescence at a magnification of ×400.

We investigated whether cell binding was modulated by the addition of free CTP1L CBD, His‐tagged at the C terminus (CBD‐His). We found that the addition of CBD‐His to binding assays with GFP‐labelled endolysins was slightly detrimental for GFP‐CTP1L and GFP‐RBSKO binding (Fig. 6). However, the binding of GFP‐CBD_D215A and GFP‐CBD_M263R and to a lesser extent GFP‐CTP1L_D215A was slightly improved after CBD‐His addition. The GFP‐CBD_T221R mutant still did not label the target cells in the presence of CBD‐His, although some protein precipitation was observed.

### Detection of C. tyrobutyricum in cheese using GFP‐CBD

**Figure 7:**
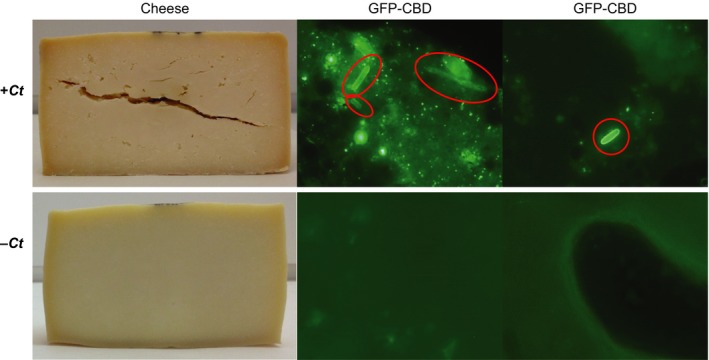
Detection of *C. tyrobutyricum* in a cheese matrix. Images of cheeses and fluorescent cells detected by GFP‐CBD in cheese with (top) or without (bottom) inoculation with *C. tyrobutyricum* INIA 68. *C. tyrobutyricum* cells are encircled; microscopy images were taken at a magnification of ×1000.

GFP‐CBD and GFP‐linker control were tested with an eight‐month‐old LBD cheese made with milk artificially contaminated with spores of *C. tyrobutyricum* INIA 68, to test direct detection of the bacterium in the cheese matrix*. *The LBD cheese contained 2.45 spores g^−1^ while no spores were detected from uninoculated cheese (detection limit 3 spores g^−1^). In agreement with the culture experiments, GFP‐CBD was able to label the vegetative cells of *C. tyrobutyricum* INIA 68, responsible for the appearance of LBD of cheese (Fig. 7), while the GFP‐linker control did not produce any fluorescence (data not shown). When the binding protocol was applied to control cheese made with the same starter cultures, MA 11 and TA 54, but without clostridial spores and without LBD, cells with green fluorescence were not observed (Fig. 7), supporting the specificity of GFP‐CBD binding.

## Discussion

*Clostridium tyrobutyricum* and related *Clostridium* spp. pose a threat to the dairy industry, causing severe economic losses due to cheese spoilage. Dormant clostridial spores may be activated during cheese manufacture and ripening, giving rise to metabolically active vegetative cells able to grow in the cheese. Cultural and culture‐independent methods for the detection of *Clostridium* spp. in cheese are available but present some disadvantages (Garde *et al*., 2013). Fluorescently labelled CBDs have been proposed as alternative tools for detection of microorganisms due to their high host‐specific binding (Bai *et al*., 2016), and they have possible application to directly detect *Clostridium* spp. in cheeses without the need for isolation.

Endolysin CTP1L consists of an EAD at the N‐terminus, encoding a glycosyl hydrolase of the GH25 type, and a CBD at the C‐terminus, with both domains connected by a short linker of four amino acid residues (Dunne *et al*., 2016). Previously, we determined that the truncated EAD of CTP1L failed to lyse *C. tyrobutyricum* NCIMB 9582 cells (Mayer *et al*., 2010); our current results confirm that the C‐terminal domain is required for the binding of CTP1L to the outside of cells, suggesting that this is necessary for lytic activity. It is known that some endolysins, including GH25‐type endolysins (Sanz *et al*., 1992; Porter *et al*., 2007; Sugahara *et al*., 2007), require a CBD for full lytic activity from without, while the EADs of other endolysins maintain or even increase activity in the absence of their CBD (Schmelcher *et al*., 2012; Rodríguez‐Rubio *et al*., 2016). The *N*‐acetylmuramoyl‐L‐alanine amidase EADs of clostridial endolysins CD27L and CS74L showed similar or increased lytic activity and the same host range as their full‐length endolysins, and GFP‐labelled CD27L EAD showed strong binding to *C. difficile* (Mayer *et al*., 2011, 2012). The ability of the EAD to act on its own may be related to its cell wall binding activity.

We evaluated the binding affinity of GFP‐labelled CTP1L and the CBD to *C. tyrobutyricum* cells at different conditions. Optimal binding was observed at pH 7, however, fluorescence intensity decreased when the pH of the buffer was lowered to 5.2. CTP1L retains lytic activity at pH 4.5 and 5.5 (Mayer *et al*., 2010), and therefore, the decrease in cell fluorescence may be due to impaired GFP fluorescence, which is influenced by changes in pH (Ward and Bokman, 1982). Cells in stationary phase were more strongly labelled by GFP‐CTP1L and GFP‐CBD than those in exponential phase. This might be due to changes in the cell wall structure that render the binding ligand more available, or to a more prevalent expression of the ligand in the stationary phase. It has recently been reported that the CBDs of *Bacillus anthracis* phage endolysins are able to recognize cell culture age‐related features on the bacterial surface (Paskaleva *et al*., 2015). Boiling *Clostridium* cells also increased the binding efficiency of CTP1L and its CBD. This might be the result of alterations to the structure of the cell wall or conformational changes in certain components on the cell surface which may increase ligand accessibility.

To broaden our understanding of the relationship between structure and function for the CTP1L CBD, we used GFP‐labelled proteins to analyse how different mutations within the CBD affected its binding ability. Shutting down the internal translation site of CTP1L to stop tCBD production (RBSKO mutant) did not prevent binding of endolysin to *C. tyrobutyricum* cell walls when in high concentrations (3 μM), indicating that the formation of an oligomer with free CBD is not essential for binding in this *in vitro* scenario. This is corroborated by the observation that the reduction in lytic activity shown by the RBSKO mutation at low (0.1 μM) lysin concentration was not apparent when high concentrations of protein were used (1 μM, Dunne *et al*., 2016). Oligomerization may have more impact on the efficacy of binding at physiological concentrations. Mutagenesis disrupting the head‐on (M263R and D215A) or side‐by‐side (D215A, T221R) dimerization interfaces abolished attachment of the endolysin to the external host cell wall, suggesting that, apart from their role in the association with other CBDs, these residues might be involved in successful binding to target ligands. The inability of these mutated endolysins to bind to cells was matched by their lack of lytic activity. However, D215A and M263R mutants were able to bind to cells which had been treated with lysozyme – this treatment might make the target ligand more accessible, or mutants might be able to bind to a different ligand than the external cell wall receptor, which became accessible after lysis. The treatment may also allow the lysin to access the wall from the inside of the cell, as it has evolved to do. In contrast, lysozyme treatment did not enable binding of the T221R mutant, indicating that this mutation has a greater impact on protein structure and/or binding.

The addition of free CBD‐His was counterproductive for the binding of the wild‐type GFP‐CTP1L and the RBSKO mutant; these results most likely reflect a competition for cell binding epitopes, as suggested previously (Dunne *et al*., 2016). The addition of CBD‐His to M263R and D215A mutants gave a limited increase in binding to the clostridial cell wall, suggesting that there had been some oligomerization with the functional CBD‐His but that either the extent of oligomerization or the structure of the final complex was not sufficient to produce significant binding. It is interesting that addition of CBD‐His to the M263R mutant, which can form strong side‐by‐side interactions, did not show any greater improvement than to the more disruptive D215A mutation. The results of the T221R mutant suggest that either this mutant cannot form oligomers with the CBD‐His or the formed complex is still unable to bind to the surface of *C*. *tyrobutyricum* cells.

GFP‐CTP1L and GFP‐CBD showed a *Clostridium*‐level of binding specificity, with GFP‐CBD showing a slightly broader range. The smaller size of GFP‐CBD could facilitate its access to target ligands within *Clostridium* cell walls. Different levels of binding specificity have been described for bacteriophage endolysins. A GFP fusion protein of CPF369_CBD, a putative CBD identified from the genome of *C. perfringens* ATCC 13124, only bound to *C. perfringens* cells (Kong and Ryu, 2016) while a GFP fusion protein of the C‐terminal domain of PlyL, an endolysin encoded by a *Bacillus anthracis* prophage, bound to *Bacillus cereus* but not to *Bacillus megaterium* or *Bacillus subtilis* cells (Low *et al*., 2005). A fusion of EGFP (enhanced GFP) with PlyG, an endolysin produced by gamma phage, or its truncated C‐terminal domains, specifically recognized *B. anthracis* cells (Yang *et al*., 2012) while an EGFP fusion protein of the C‐terminal domain of LysPBC4, the endolysin of a *B. cereus*‐specific bacteriophage, could only decorate limited strains of *B. cereus* (Na *et al*., 2016). In the case of non‐spore‐forming bacteria, GFP‐CBDs of *Listeria* phage endolysins Ply500, Ply118 and PlyP35 only bound to the *Listeria* genus and showed binding specificity at species and even serovar level (Loessner *et al*., 2002; Schmelcher *et al*., 2010). However, GFP‐CBD of Lyb5 endolysin of bacteriophage ϕPYB5, isolated from *Lactobacillus fermentum*, also bound to *L. lactis* and *Lactobacillus casei* cells (Hu *et al*., 2010) while GFP‐CBD of BFK20 endolysin from corynephage BFK20 infecting *Brevibacterium flavum* CCM 251 bound to *Brevibacterium* and *Corynebacterium* cells with different binding abilities (Gerova *et al*., 2011). GFP fusion protein of LysGH15B, a staphylococcal phage lysin, showed binding specificity to *Staphylococcus aureus* and *Staphylococcus epidermidis*, but did not bind to all staphylococcal strains tested (Gu *et al*., 2011). Similarly, GFP‐CBD of endolysin CTP1L did not bind successfully to all *Clostridium* spp. cheese isolates tested, and it will be important to ascertain whether strains which were not detected are potential causative agents of LBD, and whether modification of the labelled CBD could improve detection. This variation in binding to different species and under different conditions, together with the potential for a varied number of lysin molecules to bind to each cell, means that fluorescence detection using GFP‐labelled lysins would not be an accurate method for quantification of cells. However, specific cell wall binding domains do have the potential to improve quantification methods – Walcher *et al*. (Walcher *et al*., 2010) used paramagnetic beads coated with a CBD from a *Listeria* phage to capture *Listeria monocytogenes* from milk which was then quantified by real‐time PCR.

Although some authors (Mayer *et al*., 2010; Nakonieczna *et al*., 2015) have pointed out that endolysins may not bind to spores due to the differences in cell wall composition compared to vegetative cells, CTP1L and its CBD successfully bound to spores. This suggests that GFP‐CTP1L and GFP‐CBD could be good biomarkers for rapid detection of *Clostridium* spores in milk, so that measures can be taken for the prevention of LBD in cheese. This is the first time that an endolysin has been found to bind to *Clostridium* spores*,* and to our knowledge, there is only one previous study that describes the binding of an endolysin to bacterial spores. EGFP‐PlyG bound to spores and vegetative cells of *B. anthracis,* but the endolysin uses different domains to recognize them (Yang *et al*., 2012). Further studies are necessary to establish if CTP1L uses different domains to target spores and vegetative cells as in the case of PlyG, or whether the *C. tyrobutyricum* cell wall and spore share an epitope recognized by the CBD.

Most methods for detecting *C. tyrobutyricum* are based on spore germination and vegetative growth. GFP‐CBD allowed us to visualize directly and specifically for the first time the vegetative cells of *C. tyrobutyricum* in the matrix of a LBD cheese. In addition, GFP‐CBD specifically targeted *Clostridium* strains in diverse cellular stages and conditions. This makes GFP‐CBD a potentially beneficial agent for rapid and specific detection and tracking of *Clostridium* populations throughout cheese manufacturing (milk to whey and curd), ripening (unripe to mature cheese) and final product storage.

## Experimental procedures

### Bacterial strains and growth conditions

Strains of *Clostridium* spp. were obtained from the NCIMB (Aberdeen, UK), the ATCC (Manassas, VA, USA), the CECT (Valencia, Spain), the BCCM/LMG (Ghent, Belgium) or from the INIA in‐house collection of *Clostridium* strains isolated from Manchego cheese with LBD (Madrid, Spain, Garde *et al*., 2011, 2012). *Clostridium* strains were maintained in Robertson's cooked meat medium (SGL, Corby, UK) at room temperature and were grown anaerobically in a controlled anaerobic cabinet (A95 anaerobic workstation, Don Whitley Scientific Ltd in an atmosphere of 5% CO_2_, 10% H_2_, 85% N_2_), or in jars with an H_2_ plus CO_2_ generating kit (AnaeroGen; Oxoid, Basingstoke, UK) at 37°C in Reinforced Clostridial Medium (RCM; Oxoid) for 24–48 h. *Escherichia coli* strains (Invitrogen, Paisley, UK) were grown at 37°C with shaking in LB broth (10 g l^−1^ Bacto tryptone, 5 g l^−1^ yeast extract [Difco, Detroit, MI, USA], 10 g l^−1^ NaCl, pH 7.5) for 24 h. Lactic acid bacteria were obtained from IFR (Norwich, UK) and INIA collections. *Lactococcus lactis* strains were grown at 30°C in M17 broth (Difco) supplemented with 0.5% (wt/vol) glucose (GM17) for 24 h; *Enterococcus* spp. were grown at 37°C in brain heart infusion broth (BHI; Oxoid) for 24 h; *Lactobacillus reuteri* 1063N (Mackenzie *et al*., 2010) was grown in de Man, Rogosa and Sharpe broth (MRS; Oxoid) at 37°C for 24 h. Commercial cheese starter cultures were Choozit™ MA 11 (Danisco, Laboratorios Arroyo, Santander, Spain) containing *L. lactis* subsp. *lactis* and *L. lactis* subsp. *cremoris* strains, Choozit™ TA 54 (Danisco) containing *Streptococcus thermophilus* strains, FD‐DVS FLORA DANICA (Chr Hansen, Tres Cantos, Spain) consisting of *L. lactis* subsp. *lactis*,* L. lactis* subsp. *cremoris, L. lactis* subsp. *lactis* biovar diacetylactis and *Leuconostoc mesenteroides* subsp*. cremoris* strains and RSF 636 (Chr Hansen) formed by *L. lactis* subsp. *lactis*,* L. lactis* subsp. *cremoris, S. thermophilus* and *Lactobacillus helveticus* strains. Commercial starter cultures were suspended in sterile 0.1% (wt/vol) peptone solution (Difco) and diluted to an appropriate concentration for endolysin binding assays.

### Spore production and purification

Sporulation of seven *Clostridium* strains was induced by two different protocols. *C. tyrobutyricum* CECT 4011 and INIA 68 were grown anaerobically at 37°C in modified RCM (without agar) for 3 days. Spores/cells suspensions were centrifuged (10 000 *g,* 15 min), resuspended in 1 ml sterile milliQ water and stored at −30°C. Other clostridial strains for spore preparation were grown anaerobically in RCM broth at 37°C for 3 days then streaked onto RCM agar plates. After 3‐days incubation in anaerobic conditions at 37°C, spores/cells were collected from the plate with a swab, resuspended in 1 ml sterile milliQ water and stored at −30°C.

To separate vegetative cells and cellular debris and force the release of the spores from the mother cells, the spore/cell suspensions were subjected to a lysis protocol, modified from Yang *et al*. (2009). 1 ml spore per cell suspension was centrifuged at 10 000 *g* for 10 min at 4°C. The pellet was resuspended in 5 ml lysis buffer (1 M Tris–HCl pH 8, 0.5 M EDTA, 1 M NaCl, 0.5% N‐lauroylsarcosine sodium salt, 0.5% Brij 58, 0.2% sodium deoxycholate) containing 1 mg ml^−1^ lysozyme (Fluka, Honeywell, Bucharest, Roumania) and 20 U ml^−1^ mutanolysin (Sigma, Gillingham, Dorset, UK), incubated with gentle shaking at 37°C in a water bath for 1 h and sonicated for 10 min. These two steps were repeated once again with fresh lysis buffer. Spores were washed 10 times with sterile milliQ water. Finally, the pellet was resuspended in 100 μl PBS‐T (PBS, 0.01% Tween‐20, pH 7.4) and frozen at −30°C. *C. beijerinckii* INIA 77 was not subjected to this lysis and sonicating protocol as it was able to release the spores from the mother cells by itself. In this case, after collecting spores/cells from the streak RCM plate, the suspension was incubated overnight with lysozyme (400 μg ml^−1^; Sigma) at 42°C and spores were separated from vegetative cell debris in a Percoll^®^ gradient (Sigma) by centrifugation at 4000 *g* for 30 min at 4°C, as previously described (Leuschner *et al*., 1999). Spores, appearing at the bottom of the gradient, were harvested, washed 10 times, resuspended in sterile milliQ water and stored at −30°C until used. The presence of clean refringent spores was confirmed using phase‐contrast microscopy.

### Production of GFP‐labelled endolysins and site‐directed mutants

The full‐length endolysin CTP1L sequence is available in the ΦCTP1 nucleotide sequence under accession number HM159959, phiCTP1_gp29 (Mayer *et al*., 2010). CTP1L‐T221R and CTP1L‐D215A were described previously (Dunne *et al*., 2014); CTP1L‐M263R was made in the same way using the Quikchange method (Stratagene, Waldbronn, Germany) and primers M263R_F and M263R_R (Table S1). Translational fusions of GFP and CTP1L were created by splice overlap extension PCR (Horton *et al*., 1989). PCR was performed in two steps using Phusion polymerase (Finnzymes, New England Biolabs, Hitchin, UK) and primers listed in Table S1. The GFP gene and a flexible linker were amplified from construct *gfp‐linker*‐pET15b (Mayer *et al*., 2011) using primer pET15_F, located in the pET15b vector upstream of the NdeI cloning site and splice primer GFPspliceCTP1L_R, consisting of 15 nt to match the end of the linker and 17 nt (underlined) to match the start of the CTP1L gene. The CTP1L gene was amplified from the construct *ctp1 l‐*pET15b (Mayer *et al*., 2010) using primer pET15_R located in the pET15b vector downstream of the BamHI‐HF cloning site and splice primer CTP1LspliceGFP_F consisting of 16 nt to match the GFP linker followed by 17 nt to match the start of the CTP1L gene (underlined). The CTP1L N‐terminal domain (EAD) was amplified from a construct containing CTP1L truncated by a stop codon after the Asn197 codon cloned into pET15b (Mayer *et al*., 2010) using pET15_R and CTP1LspliceGFP_F. Primary PCR products were then spliced to create hybrid genes using outer primers pET15_F and pET15_R and annealing conditions calculated to allow the overlapping central portions to anneal. The spliced products were restricted with NdeI and BamHI‐HF (New England Biolabs) and cloned into pET15b (Novagen, Merck, Nottingham, UK) similarly restricted then dephosphorylated with Antarctic Phosphatase (New England Biolabs), using Fastlink ligase (Epicentre, Cambio, Cambridge, UK). Ligation products were transformed into chemically competent *E. coli* TOP10 cells (Invitrogen) or electrocompetent *E. coli* DH5α, and transformants were selected with ampicillin (100 μg ml^−1^). Inserts were confirmed by sequencing, and constructs were transformed into electrocompetent *E. coli* BL21 (DE3) (Invitrogen) for expression.

GFP‐CBD_D215A, GFP‐CBD_T221R, GFP‐CBD_M263R, GFP‐CTP1L_D215A and GFP‐RBSKO mutants (Fig. 1A) were made in the same way using splice overlap PCR. GFP‐CBD_D215A, GFP‐CBD_T221R, GFP‐CBD_M263R were created by amplification of two products from *gfp‐ctp1lcbd*‐pET15b (Dunne *et al*., 2016) using primers to insert the relevant mutation combined with pET15_F (D215A_R, T221R_R and M263R_R, respectively) and pET15_R (D215A_F, T221R_F and M263R_F, respectively). GFP‐CTP1L_D215A was prepared in the same way but from construct *gfp‐ctp1l*‐pET15b. Product pairs were then spliced, restricted and subcloned as before. GFP‐RBSKO was prepared from two sets of splice overlap reactions. Plasmid *ctp1lSSSS*‐pET15b (Dunne *et al*., 2016) was used with pET15_F and SSspliceCTP1L_R and *ctp1l*‐pET15b was used with pET15_R and CTP1LspliceSS_F; splicing these products recreated the RBSKO sequence K190S_G191S. The mutant RBSKO‐CTP1L sequence was then spliced to GFP in the same way as the wild‐type CTP1L as described above.

### Protein expression and analysis

The expression of GFP‐labelled proteins in *E. coli* BL21 (DE3) was induced with 0.5 mM IPTG (isopropyl‐β‐D‐thiogalactopyranoside, Melford Laboratories, Ipswich, UK). Crude protein lysates from induced cultures were produced by cell disruption as described previously (Mayer *et al*., 2008). His‐tagged endolysins were partially purified under native conditions using nickel‐nitrilotriacetic acid (Ni‐NTA) agarose (Qiagen, Manchester, UK); proteins were recovered in elution buffer (10 mM Tris–HCl, 150 mM NaCl, 200 mM imidazole, pH 8.0). Protein production was routinely induced for 4 h at 37°C, but GFP‐CTP1L_D215A was induced overnight at 21°C to improve yield. Proteins were quantified using Bradford reagent (Bio‐Rad Laboratories, Perth, UK) and visualized on 10% NuPage Novex bis‐Tris gels in MES (2‐(N‐morpholino) ethanesulfonic acid) buffer, stained with Simply Blue Safestain (Invitrogen). Lytic activity was measured by turbidity reduction assays using frozen cells with 1 μM endolysin as described previously (Mayer *et al*., 2010).

Binding of GFP‐labelled proteins was first tested with *C. tyrobutyricum* NCIMB 9582 as before (Loessner *et al*., 2002; Mayer *et al*., 2011) with some modifications. In brief, bacterial cells were grown for 24 h in their optimal medium and conditions. Cells were harvested by centrifugation (13 000 *g*, 2 min), resuspended in 1/10 volume of PBS‐T and stored on ice. Subsequently, equal volumes of cells and GFP‐labelled endolysins or GFP‐linker negative control at a final concentration of 3.0 μM protein were mixed and incubated at 25, 30 or 37°C for 5, 10 or 20 min at pH 7.4 (PBS‐T) or 5.2 (PBS‐T adjusted with 1 M HCl). Different concentrations of NaCl (2, 1, 0.5, 0.2 M) were also tested. Cells were then removed from the supernatant by centrifugation (13 000 *g*, 2 min), washed twice with 200 μl PBS‐T and the pellet was resuspended in 10 μl PBS‐T buffer for fluorescence microscopy. Binding assays were also carried out with cells in the log (OD_600_ 1) or stationary (OD_600_ 2–3) phase, and with cells that had been boiled (100°C for 10 min) or treated with lysozyme (1 mg ml^−1^, 37°C, 3.5 h). Samples were viewed with a Nikon Eclipse 50i microscope equipped with a LED illumination unit pE‐300^white^, DS Camera Head DS‐Fi1 and NIS‐Elements 2.34× version imaging software. Fluorescence was excited with an LED light with excitation and emission filters for GFP detection (495 and 530 nm).

The spectrum of binding activity of GFP‐CTP1L, GFP‐EAD and GFP‐CBD was tested at the optimal binding conditions (37°C, 20 min in PBS‐T buffer) with different *C. tyrobutyricum, C. sporogenes, C. beijerinckii* and *C. butyricum* strains, lactic acid bacteria and frozen spore stocks. Binding assays with GFP‐CBD_D215A, GFP‐CBD_T221R, GFP‐CBD_M263R, GFP‐CTP1L_D215A and GFP‐RBSKO mutants were carried out only with *C. tyrobutyricum* NCIMB 9582 with optimal binding conditions. To examine free CBD addition, GFP‐labelled proteins were mixed with CBD‐His (Dunne *et al*., 2016) at a molar ratio of 1:10 prior to binding.

### Detection of *C. tyrobutyricum* labelled with GFP‐CBD within the matrix of a LBD cheese

An eight‐month‐old cheese made with milk artificially contaminated with 1 × 10^5^ spores of *C. tyrobutyricum* INIA 68 per ml and showing LBD symptoms was used for the detection of *Clostridium* directly in a cheese matrix. A cheese made with the same starter culture but without clostridial spores served as control. The method of Fernández de Palencia *et al*. (2004) was followed with some modifications. Briefly, cheese (0.4 g) was homogenized with 1 ml PBS buffer (pH 8), incubated for 5 min at 30°C, centrifuged (6000 *g,* 10 min) and washed twice in PBS buffer. Then, the protocol followed for binding assays was similar to that described above – approximately 50 μl of the cheese sample was mixed with the GFP‐linker control or with GFP‐CBD to a final concentration of 3.0 μM protein, incubated at 37°C for 20 min and washed twice with PBS buffer. Finally, a small piece of cheese sample was spread along a glass slide for fluorescence microscopy. Lactate fermenting *Clostridium* spores were enumerated from homogenized cheese samples (10 g) as described previously (Garde *et al*., 2011).

## Conflict of interest

None declared.

## Supporting information


**Fig. S1.** Increase in binding of GFP‐CBD to boiled cells. Microscopy images were taken with bright field and fluorescence at a magnification of ×400 and at equivalent exposures.

**Table S1.** Specific primers pairs used for splice overlap extension PCR, site‐directed mutagenesis and analysis of CTP1L constructs^a^
