# Supplementary figures and images for: Development of a specific fluorescent phage endolysin for in situ detection of Clostridium species associated with cheese spoilage
Source: Microb Biotechnol. 2017 Nov 21;11(2):332–45. doi: 10.1111/1751-7915.12883 (PMC5812242; doi:10.1111/1751-7915.12883)

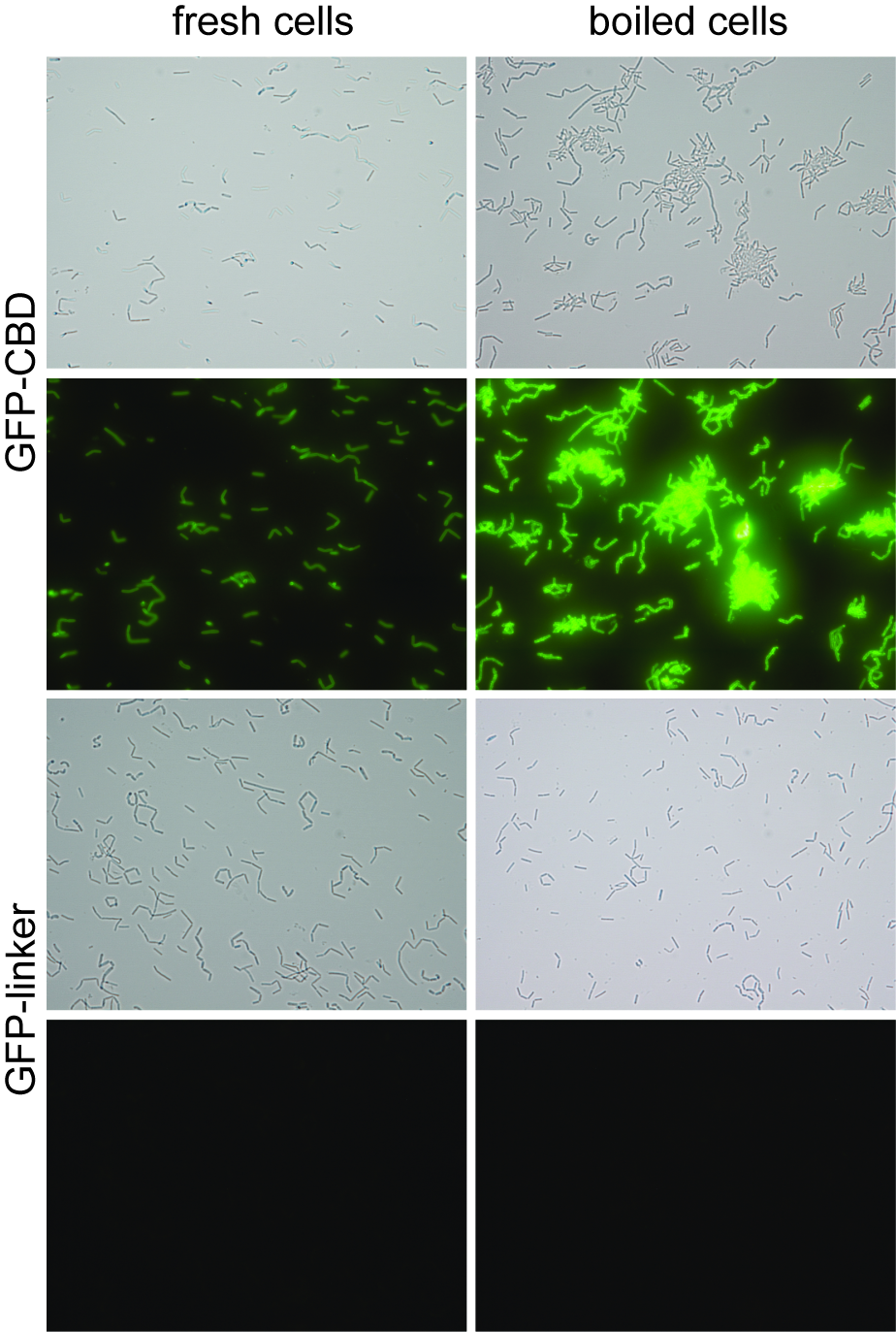

Supplement: Supplementary file 1 — Fig. S1. Increase in binding of GFP‐CBD to boiled cells. Microscopy images were taken with bright field and fluorescence at a magnification of ×400 and at equivalent exposures. [file MBT2-11-332-s001.tif]
