# Supplementary material for: Development of a specific fluorescent phage endolysin for in situ detection of Clostridium species associated with cheese spoilage
Source: Microb Biotechnol. 2017 Nov 21;11(2):332–45. doi: 10.1111/1751-7915.12883 (PMC5812242; doi:10.1111/1751-7915.12883)
Supplement: Supplementary file 2 — Table S1. Specific primers pairs used for splice overlap extension PCR, site‐directed mutagenesis and analysis of CTP1L constructsa [file MBT2-11-332-s002.docx]

**Table S1** Primers pairs used for splicing overlap extension PCR, site-directed mutagenesis and analysis of CTP1L constructs^a^

Primer Nucleotide sequence

M263R_F 5'-gctcaacaaggtacacaactaggcaggcagtact-3'

M263R_R 5’-cgagttgttccatgtgttgatccgtccgtcatga-3’

GFPspliceCTP1L_R 5’-TCTGCTATTTTCTTCATTCCACTACCTGATCC-3’

CTP1LspliceGFP_F 5’-TGGATCAGGTAGTGGA**ATG**GAAAATTTAGTAGTTTAT-3’

pET15_F 5’-CATCATCATCACAGCAGCG-3’

pET15_R 5’-GCAGCCAACTCAGCTTCC-3’

D215A_F 5’-TTAGCAGCTAGATTGGCATGT-3’

D215A_R 5’-GCCAATCTAGCTGCTAAATATTCT-3’

T221R_F 5’-CATGTCCACGTATTAACAATG-3’

T221R_R 5’-TGTTAATACGTGGACATGCCAAT-3’

M263_F 5’-CACAACTAGGCAGGCAGTACTT-3’

M263_R 5’-CTGCCTGCCTAGTTGTGTACCT-3’

SSspliceCTP1L_R 5’-TTCCACTTCATCTTCAGAAGAAATATATTTTATAAATTC-3’

CTP1LspliceSS_F 5’-TAAAATATATTTCTTCTGAAGATGAAGTGGAAAAT-3’

T7P2_F 5’-TGAGCGGATAACAATTCCC-3’

T7T_R 5’-GCTAGTTATTGCTCAGCGG-3’

^a^ Overlap sequences are underlined and start site is in bold.
